# mNG-tagged *mls-2* *knock-in* alleles in *C. elegans*

**DOI:** 10.17912/micropub.biology.000529

**Published:** 2022-02-22

**Authors:** Rui Xiong, Yi-Wen Hsieh, Chiou-Fen Chuang

**Affiliations:** 1 Department of Biological Sciences, University of Illinois at Chicago; 2 Graduate Program in Neuroscience, University of Illinois at Chicago

## Abstract

The *Caenorhabditis elegans* HMX/NKX MLS-2 transcription factor was previously shown to play sequential roles in AWC general identity and the stochastic AWC^ON^/AWC^OFF^ subtype choice during embryogenesis. Here we analyze the expression pattern of endogenous *mls-2 *during AWC development using mNeonGreen (mNG) knock-in strains. Similar to transgenic GFP::MLS-2, functional mNG::MLS-2 knock-in displayed nuclear localization in AWC precursor cells but was not observed in AWC during the later embryonic stage. These results suggest that the expression of *mls-2* is below the detectable level and/or the stability of MLS-2 protein is very low in AWC cells.

**Figure 1. Expression patterns of mls-2p::GFP::mls-2 transgene, mNG::SEC::mls-2 knock-in, and mNG::mls-2 knock-in:**
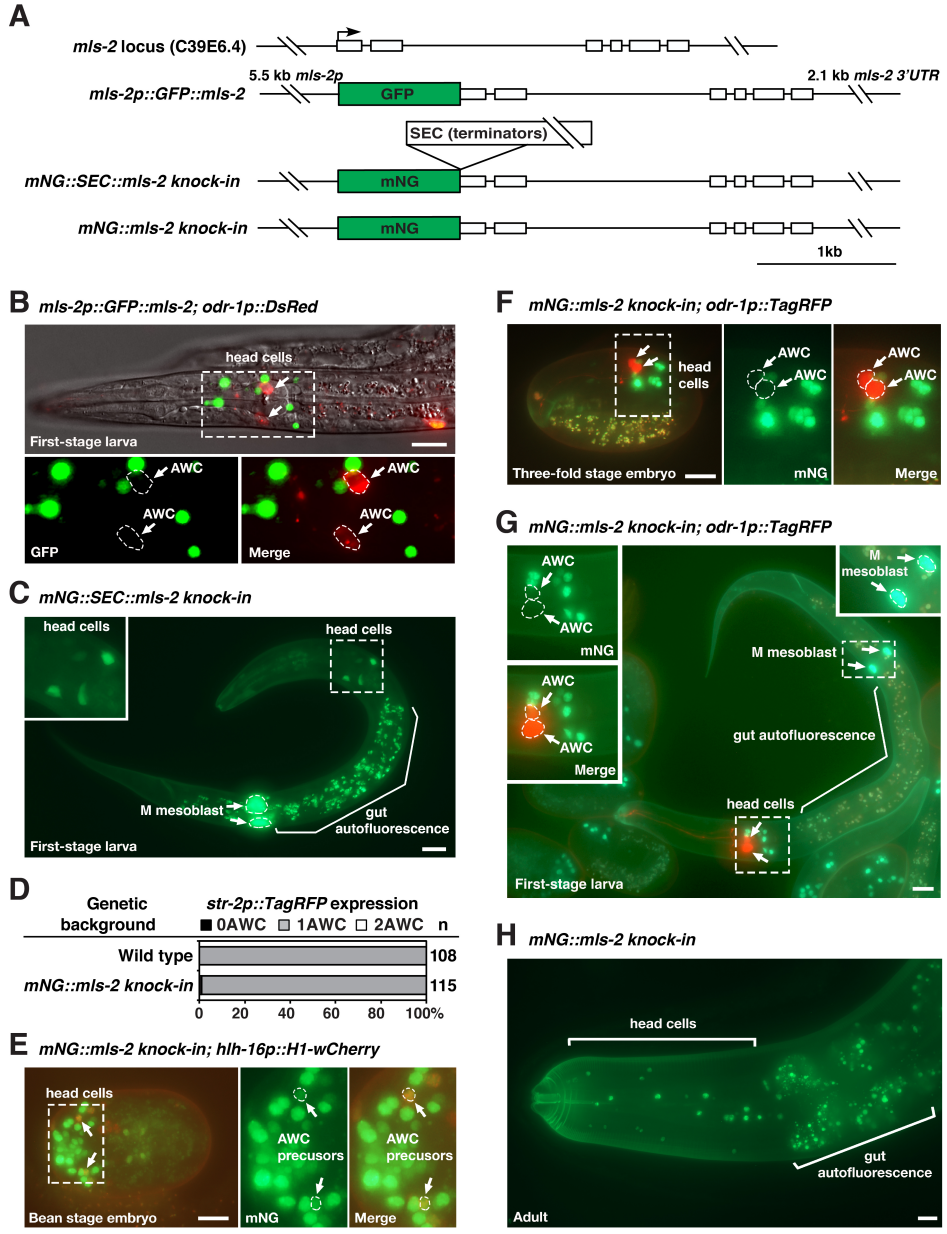
(**A**) Structure of the *mls-2* locus, *mls-2p::GFP:*:*mls-2* transgene (Jiang *et al.*, 2005), *mNG::SEC::mls-2 knock-in*, and *mNG::mls-2* *knock-in*. mNG, mNeonGreen. SEC, self-excising cassette containing transcriptional terminators, a dominant roller phenotype marker *sqt-1(e1350),* Cre driven by a heat shock promoter, and a hygromycin resistance gene. The SEC cassette is flanked by LoxP sites. (**B-H**) Representative images of GFP::MLS-2 expression from a *mls-2p::GFP::mls-2* extrachromosomal transgene in a first-stage larva (B), mNG expression from *mNG::SEC::mls-2 knock-in* in a first-stage larva (C), and mNG::MLS-2 expression from *mNG::mls-2 knock-in* animals in different developmental stages (E-H). Integrated transgenes of *hlh-16::H1-wCherry* and *odr-1p::TagRFP* (or *odr-1p::DsRed*) were used as early and late AWC markers, respectively. Insets in panels B, C, E, F, and G are magnified by 2-fold. Scale bar, 10 um. Anterior to the left and ventral to the bottom in lateral or ventrolateral views of the head region in B, C, and F-H; ventral view in E. (D) Expression of the AWC^ON^ marker *str-2p::TagRFP* from an integrated transgene in wild type and *mNG::mls-2* *knock-in* animals. n, total number of animals scored.

## Description

The HMX/NKX MLS-2 transcription factor plays a role in the development of the postembryonic mesoderm, CEPsh glia, tube cells of the excretory system, general AWC identity, and AWC asymmetry in *C. elegans* (Jiang *et al.*, 2005; Yoshimura *et al.*, 2008; Kim *et al.*, 2010; Abdus-Saboor *et al.*, 2012; Hsieh *et al.*, 2021)*.* The expression pattern of MLS-2 protein has been previously examined by immunohistochemical staining with anti-MLS-2 antibodies, transgenes of GFP-tagged MLS-2 (GFP::MLS-2), and *mls-2*::GFP fosmid reporter lines (Jiang *et al.*, 2005; Yoshimura *et al.*, 2008; Kim *et al.*, 2010; Abdus-Saboor *et al.*, 2012; Walton *et al.*, 2015; Reilly *et al.*, 2020) (Figure 1A). It was shown that GFP::MLS-2 was expressed in the embryonic AWC lineages from automated lineage analysis and was detected transiently in AWC neurons in first-stage larvae (Kim *et al.*, 2010; Abdus-Saboor *et al.*, 2012; Walton *et al.*, 2015). However, we did not detect expression of GFP::MLS-2 transgenes in AWC neurons in late embryos or the first larval stage (Figure 1B).

To determine the expression pattern of endogenous *mls-2* locus in AWC, we generated *mNG::SEC::mls-2 knock-in* and *mNG::mls-2 knock-in* animals by tagging the 5’ end of endogenous *mls-2* coding region with mNG::SEC or mNG using Cas9-triggered homologous recombination (Dickinson *et al.*, 2013; Dickinson *et al.*, 2015; Dickinson and Goldstein, 2016) (Figure 1A). The *mNG::SEC::mls-2 knock-in* allele is a transcriptional reporter of *mls-2*, since the self-excising cassette (SEC) contains transcriptional terminators. *mNG::SEC::mls-2* *knock-in* showed diffuse mNG expression in numerous cells in the head and the M mesoblast of first-stage larvae (Figure 1C). The *mNG::mls-2 knock-in* allele, generated by the SEC excision of *mNG::SEC::mls-2* *knock-in*, is a translational reporter of MLS-2 protein. *mNG::mls-2 knock-in* animals displayed wild-type AWC asymmetry as determined by the expression of the AWC^ON^ marker *str-2p::TagRFP* (Figure 1D), suggesting that mNG::MLS-2 fusion protein is functional in AWC development. Like GFP::MLS-2 expressed from transgenes, mNG::MLS-2 knock-in was localized in the nucleus of AWC precursor cells in early embryos (Figure 1E) but was not observed in AWC cells in late embryos (Figure 1F) or early-stage larvae (Figure 1G). Our results are consistent with single-cell RNA-seq data showing that *mls-2* was briefly expressed at a very low level in AWC during early embryogenesis but not detected in AWC in second-stage larvae (Cao *et al.*, 2017; Packer *et al.*, 2019). It was also shown that MLS-2::GFP expressed from an integrated *mls-2*::GFP fosmid reporter line was not detected in AWC in late larval stage or young adult-stage using NeuroPAL (Reilly *et al.*, 2020).

Similar to MLS-2 antibody staining and GFP::MLS-2 transgenes (Jiang *et al.*, 2005), mNG::MLS-2 knock-in was localized to the nucleus of a subset of head cells and the M mesoblast in first-stage larvae and adults (Figure 1G and 1H). *mNG::SEC::mls-2 knock-in* and *mNG::mls-2 knock-in* strains should help to determine the endogenous expression pattern of *mls-2* in different cells during development.

## Methods

*mNG::SEC::mls-2* and *mNG::mls-2 knock-in* were generated using the Cas9-triggered homologous recombination protocol as previously described (Dickinson *et al.*, 2013; Dickinson *et al.*, 2015).

## Reagents

**Table:** 

Strain	Genotype	Source
IX1119	*oyIs44 [odr-1p::DsRed; lin-15(+)]* V*; vyEx535 [mls-2p::GFP::mls-2::mls-2 3’UTR* (Jiang *et al.*, 2005)*; ofm-1p::DsRed]*	This study
IX4507	*mls-2(vy247 [mNG::SEC::mls-2 knock-in])* X	This study
IX4506	*mls-2(vy248 [mNG::mls-2 knock-in])* X	This study
RW10588	*unc-119(ed3); zuIs178 [his-72(1kb 5′ UTR)::his-72::SRPVAT::GFP::his-72 (1KB 3′ UTR) + 5.7 kb XbaI-HindIII unc-119(+)]; stIs10544 [hlh-16::H1-wCherry::let-858 3′ UTR]*	Murray *et al.*, 2012
IX5609	*stIs10544 [hlh-16::H1-wCherry::let-858 3′ UTR]* (Murray *et al.*, 2012)*; mls-2(vy248 [mNG::mls-2 knock-in])* X	This study
IX4894	*vyIs56 [odr-1p::TagRFP]* III (Cochella *et al.*, 2014)*; mls-2(vy248 [mNG::mls-2 knock-in]) X*	This study
IX3212	*vyIs68 [str-2p::TagRFP; srsx-3p::GFP]* III	Cochella *et al.*, 2014
